# Coping and wellbeing in bereavement: two core outcomes for evaluating bereavement support in palliative care

**DOI:** 10.1186/s12904-020-0532-4

**Published:** 2020-03-12

**Authors:** Emily Harrop, Hannah Scott, Stephanie Sivell, Kathy Seddon, Jim Fitzgibbon, Fiona Morgan, Sara Pickett, Anthony Byrne, Annmarie Nelson, Mirella Longo

**Affiliations:** 1 Marie Curie Research Centre, Division of Population Medicine, Cardiff University, 8th Floor Neuadd Meirionydd, Heath Park Way, Cardiff, CF14 4YS UK; 2 School of Dentistry, Cardiff University, University Hospital Wales, Heath Park, Cardiff, CF14 4XY UK; 3 Swansea Centre for Health Economics, Swansea University, Singleton Park, Swansea, SA2 8PP UK

**Keywords:** Bereavement, Palliative care, Core outcome set, Delphi survey

## Abstract

**Background:**

Bereavement support is a core part of palliative care. However, the evidence base is limited by a lack of consistency in the outcomes used to evaluate services and models of support, which makes it difficult to compare approaches. Core Outcome Sets (COS) represent the minimum that should be measured in research into specific conditions or services. The aim of this study was to use a stakeholders’ perspective to develop a COS for evaluating bereavement support for adults in adult palliative care settings.

**Methods:**

A list of outcomes relevant to bereavement support was created following a systematic review of the quantitative and qualitative literature. At an expert workshop 21 stakeholders discussed their views on the most important outcomes and compared these to and critiqued the lists constructed from the review. These lists and discussions informed a two round international DELPHI survey (*n* = 240) designed to reach consensus on which outcomes/outcome dimensions should be included in the COS. To prioritise and validate the items emerging from the survey, participants at a subsequent consensus day ranked the relative importance of these items (*n* = 23). A final feedback exercise with these consensus day participants was conducted to confirm the selection of outcomes and dimensions.

**Results:**

‘Ability to cope with grief’ and ‘Quality of life and mental wellbeing’ were selected as two core outcomes. Twenty-one different dimensions to explore when assessing these outcomes were also identified. The coping related dimensions have been categorised as: Negative and overwhelming grief; Communication and connectedness; Understanding, accepting and finding meaning in grief; Finding balance between grief and life going forwards; Accessing appropriate support. Those relating to quality of life and wellbeing have been categorised as; Participation in work and/or regular activities; Relationships and social functioning; Positive mental wellbeing and Negative mental and emotional state.

**Conclusion:**

This COS outlines a more consistent way forward for bereavement researchers and practitioners, whilst also orientating towards public health and resilience-based approaches to bereavement care. Further work is planned to identify and develop measures which are specific to this core outcome set, and which will facilitate the future comparability of bereavement services and interventions.

## Background

Although grief is a natural process, in which many people adjust with support from their social networks, others require more formal forms of professional support [1, 2]. Bereavement is associated with elevated risks to mental health, morbidity and mortality [2–5], and services that provide bereavement support can be important for managing these risks [2]. The socio-economic costs of bereavement can also be considerable, affecting all sectors of society [6–8]. Bereavement support is an important part of palliative and end of life care, with different levels of provision recommended to meet the varying needs of bereaved individuals [1, 2, 9–11]. The public health approach to bereavement care [12, 13] and the UK National Institute for Health and Clinical Excellence (NICE) [10] both propose three tiers of bereavement support, to be made available according to level of need:

- Component 1 where information is universally offered regarding the experience of bereavement and people are sign-posted towards further support if needed. Informal support is also provided by existing social networks.
- Component 2 which makes provision for people with moderate needs to access formal opportunities to reflect upon their grief and may involve individual or mutually supportive group sessions.
- Component 3 which encompasses specialist interventions such as mental health services, psychological support and specialist counselling for those identified as having complex needs and at high risk of Prolonged Grief Disorder (PGD).

However, the research evidence available for these different types of support is limited. Inconclusive results and lack of positive effects are commonly reported in systematic reviews and meta-analyses of bereavement intervention studies [4, 14–16], with some indicating that grief interventions may only be effective for those with more severe and complicated grief symptoms [4, 16]. As also noted, however, such findings run counter to the professional experience of many clinicians in the field [15] and are challenged by the positive impacts that have been consistently reported in qualitative and mixed methods evaluations of bereavement interventions [14].

Several of these systematic reviews have identified problems with inconsistent outcome measurement [4, 14, 17–19]. A systematic review of the end of life and bereavement experiences of family caregivers identified 89 unique instruments, almost half of which were study specific with no psychometric testing reported [19]. A meta-analysis of the prevention and treatment of complicated grief noted the limitations caused by variation in outcome measures [4]; conclusions also mirrored in systematic reviews of the evidence for bereavement support generally [18], and in cancer and palliative care specifically [14, 17]. Some commentators have also suggested that bereavement intervention studies may fail to find an effect because they are measuring the wrong outcomes, often relying on narrowly defined and simplistic criteria such as psychiatric symptom checklists or global measures of functioning, which are not specific to bereavement [15]. These problems with outcome measurement undermine the potential for study results to be combined and compared, and the kind of robust conclusions on effectiveness that are needed to inform clinical practice and service delivery [14, 17, 18, 20]. Recent national and international programmes of work have focused on developing sets of service standards through consensus building activities with expert stakeholders [10, 21] (https://www.eapcnet.eu/eapc-groups/task-forces/bereavement). By reaching agreement on what constitutes a good service, and commitment amongst service providers to adhere to such standards, improved outcomes for service users should be expected to follow. However, without also identifying what these outcomes should be, the impact of these services on their service users will remain difficult to determine.

The challenge of consistent and appropriate outcome measurement is not unique to this field and in recent years consensus building methodologies have been used with expert communities to establish standardised sets of outcomes known as ‘core outcome sets’. A Core Outcome Set (COS) can be defined as an agreed minimum set of outcomes that ‘should be measured and reported in all clinical trials of a specific condition’, based on some level of stakeholder agreement over what outcomes are essential to measure (www.comet-initiative.org). A COS can improve consistency between studies and offers the following benefits: reduced heterogeneity and the ability to facilitate meta-analysis, reduced risk of reporting bias and ensuring that all trials contribute outcome data to meta-analysis [22]. By engaging with a range of stakeholders in the process it is also more likely relevant outcomes will be captured [22]. To address the limitations and gaps highlighted in the above literature, the current study aimed to develop a COS specific to bereavement research and clinical practice in palliative care. The scope of the COS was defined to include bereavement interventions or services for adults who have lost other adults to terminal illness.

## Methods

**Fig. 1:**
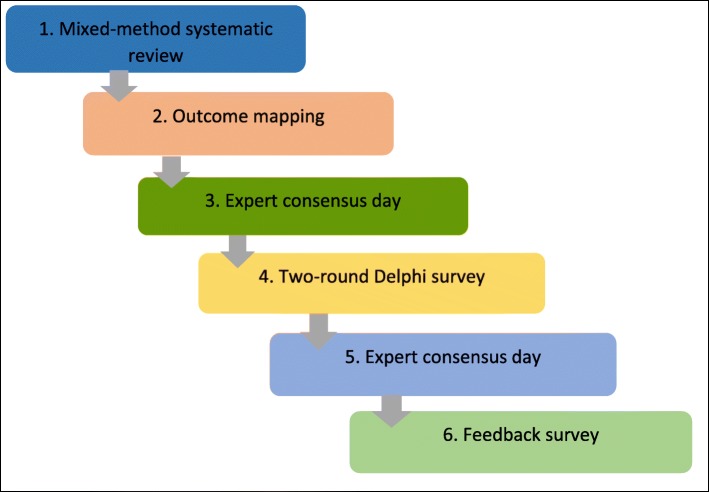
COS methodology used in study

There are a variety of methods that can be used to develop a COS, with literature or systematic reviews and Delphi techniques having become popular of late [20, 23]. This COS was developed using information on outcomes and measures collected from a systematic review, two expert consensus meetings and a modified two-round Delphi survey, to gain agreement amongst stakeholders on which outcomes and dimensions are ‘core’ (Fig. 1).

### Systematic review and outcome mapping exercise

A mixed methods systematic review of bereavement support interventions for adults bereaved through terminal illness was conducted to identify relevant outcomes and outcome measures. Specialist databases Medline, Embase, PsycINFO and CINAHL were searched using index terms and key words. A set of bereavement/grief terms were identified and combined with a set of palliative care/advanced illness/caregiver terms. Studies were included if they reported results from evaluations of bereavement support interventions or services for adults bereaved through terminal illness, delivered in ‘western’ countries and published in English from 1996 onwards. Relevant papers were identified by two independent reviewers, following a process of title, abstract and full paper screening. The protocol for this review, including full search strategy is published on Prospero (*www.crd.york.ac.uk/prospero**,* CRD42016043530). Using the same databases an additional search was carried out to identify systematic reviews of adult bereavement interventions and tools used in bereavement research and practice. This supplementary search allowed us to capture outcomes and measures that may not have been used in solely palliative care related interventions.

Once relevant studies were identified a cross tabulated Excel spreadsheet was constructed listing all outcomes used in quantitative evaluations of bereavement support, published from 1996 onwards. Two researchers (EH and SS) independently interrogated the long list of outcomes with the goal of clustering and identifying single definitions for similarly described outcomes and organising the outcomes into broader domains. Uncertainties or disagreements were resolved by discussion with the wider team. Information on the psychometric properties (outcome dimensions) of all measures used in these studies was collated and similarly described dimensions were grouped together and given single definitions following the same process. Additional measures were also included following the recommendations of other researchers or identification in supplementary searches (see additional file one: list of all measures used). Interventional impacts identified and described in the synthesis of qualitative evaluation studies, and all aspects of caregiver grief and coping experiences reported in qualitative observational studies were extracted, organised under thematic headings and described as potential outcome dimensions. These were mapped across to the lists of outcomes and dimensions created from the quantitative measures, highlighting those which ‘matched’ and adding new dimensions where needed. At the end of this process there were 11 outcomes, and 105 different outcome dimensions associated with these outcomes.

### Expert workshop

**Fig. 2:**
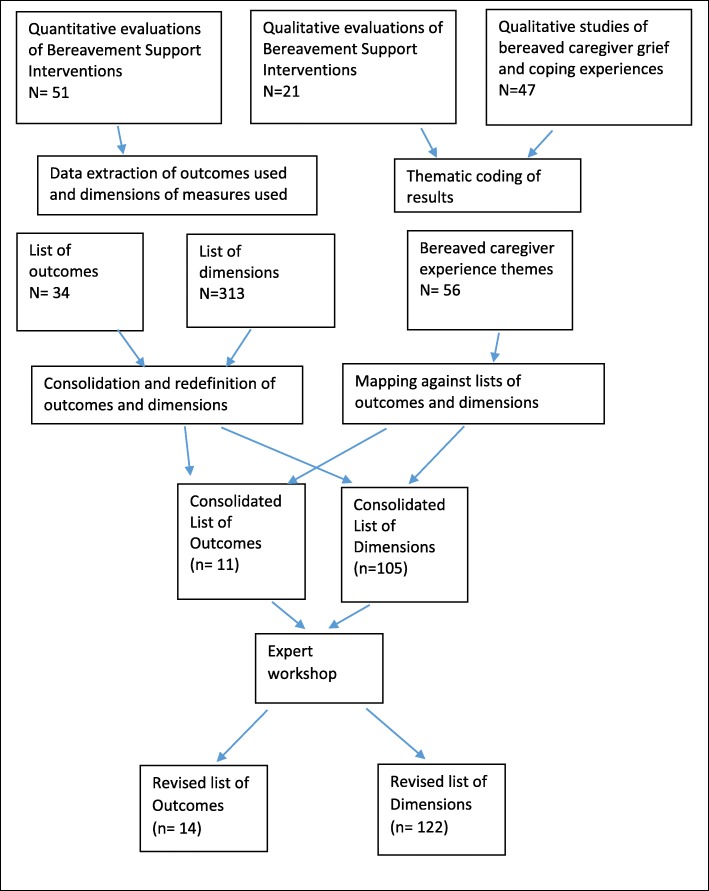
Flow diagram of outcome mapping process

To ensure that we captured all potentially relevant outcomes and outcome dimensions, a stakeholder workshop was held with 21 UK based delegates, representing a variety of professional and non-professional backgrounds. Delegates were identified through local and national bereavement provider networks and organisations, PPI networks, professional contacts and further recommendations (See Table 1 for participant details). The day was organised around breakout sessions, with two professional groups and one group of people with caregiving and bereavement experiences. In the first group session participants were asked to identify and categorise potential outcomes and outcome dimensions that they felt were important to capture when assessing how well a bereavement support service is working. In the second session each group was given a copy of the outcome lists from the systematic review and asked to discuss and critique these with reference to their suggestions from the first sessions. Following review of the points raised in the two sessions, the outcome lists used on the day were adapted, with various new items added to the lists (Fig. 2).

### Delphi survey

A modified two round Delphi survey was conducted to capture the views of stakeholders on the most important outcomes and outcome dimensions to include in the COS. A Delphi process is a systematic, interactive method using a panel of experts answering questions in two or more rounds to reach agreement on what to include in a target end product [24, 25]. The outcome lists developed from the literature and consensus day discussions were used to create the items included in the Delphi survey, following processes of further mapping, consolidation and piloting with a mix of stakeholder participants (*n* = 23). A paper and an online version of the questionnaire were produced. There were two rounds to the Delphi. In the first-round participants rated the extent to which they agreed or disagreed that each outcome/outcome dimension should be included in the core outcome set (i.e. what to measure). Participants rated the items on 5-point Likert type scales ranging from ‘not important’ to ‘very important’.

The first round of the survey remained open for 34 weeks and the second round for 4 weeks. A link to the online version of the questionnaire was sent to the corresponding authors of the studies identified in the systematic review and to participants who attended the first consensus day. These participants were also asked to circulate amongst their networks and colleagues and social media were used to promote the survey further. To increase service user participation in the survey we also invited all the UK Marie Curie Hospices to recruit current bereavement service users into the study. The bereavement research lead at each hospice identified suitable staff members and eligible service users, introduced the study and provided them with an information pack containing the participant information sheet and a paper copy of the questionnaire.

Online responses were downloaded into SPSS and paper questionnaires were manually inputted. Respondents were grouped into four key stakeholder groups: service users, service providers, members of the public and researchers. Members of the public included adults bereaved with no experience of using bereavement support services. Summary statistics (frequencies; descriptives) were run to determine consensus (defined as greater than 70% agreement) at each stakeholder’s group level. Services users’ preferences were considered top priority. As a result, an item reached consensus in the first-round if at least 70% of the overall sample and 70% of service users considered it to be ‘not important’/‘slightly important’ or ‘important’/‘very important’.

**Fig. 3:**
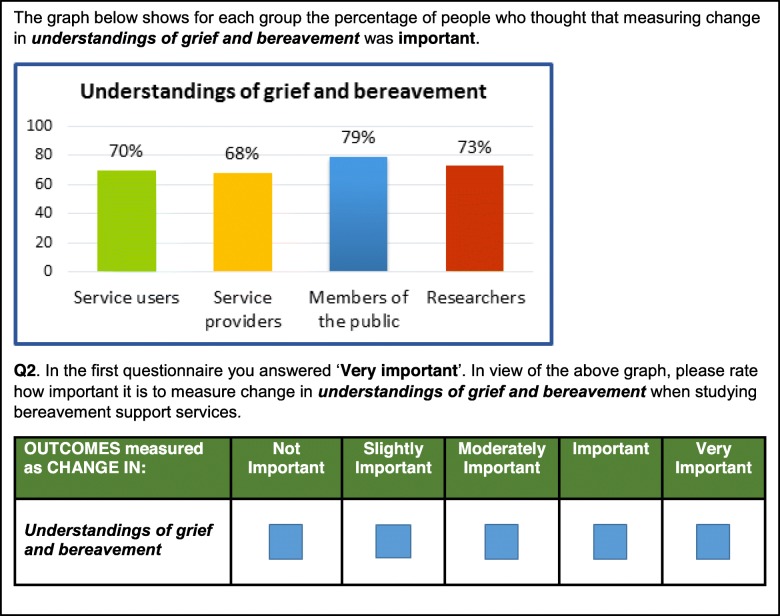
Example question used for Delphi – round 2

In round two, participants from round one were asked to re-evaluate those items which did not reach consensus. Each question included a graph to show the percentage of agreement reached across each stakeholder group, a reminder of the answer the respondent provided in round one and a question asking to indicate their preferences again (Fig. 3).

### Final consensus day

A final consensus day was held in April 2018 to help prioritise and validate the selection of items emerging from the survey. Delegates were presented with a series of lists of outcomes and outcome dimensions. In order to reduce burden on the respondent, each question included up to seven items and participants were allowed up to three votes depending on the scores that the items had reached in the survey. Using electronic voting technology participants voted on what they felt were the most important items. After voting for each question, the participants were presented with the survey results and the two sets of results were discussed.

In the second session, the outcomes and outcome dimensions that were shortlisted were presented and participants were asked to check that they agreed with the items that were shortlisted and identify any items not on the shortlists that they felt should be included. Unfortunately, due to time constraints it was not possible to satisfactorily complete this exercise. It was thus agreed that these sets of results would be circulated to provide participants with the opportunity to consider and provide feedback on the emergent selection of outcomes and dimensions.

### Mapping exercise and feedback survey

The results from the Delphi rounds and voting exercises demonstrated good consistency between the highest scoring outcomes, with a clear indication for the first core outcome. To inform and further validate the selection of other potential core outcomes, a mapping exercise was conducted which re-connected the highest scoring outcome dimensions from the Delphi survey with their associated outcomes. The results of this exercise demonstrated that almost all of these outcome dimensions mapped back to the top three outcomes from the Delphi survey. However, it also showed significant duplication between the dimensions associated with the second and third outcomes, suggesting a need to choose between these two outcomes.

A feedback survey detailing results from the Delphi survey and consensus day voting was sent to all consensus day delegates, highlighting convergent and divergent items. Based on these results respondents were asked to describe their preferences for the uncertain second outcome and to identify any excluded outcome dimensions that they felt should be included in the COS. The dimensions proposed for exclusion were those that scored below 80% in the Delphi survey in both the overall sample and the service user only sample. It was decided that an excluded dimension could be added to the final version of the COS if a clear majority (80% or more) of respondents in the final feedback survey selected it. If there was no majority the item would remain excluded, and in cases where the majority was marginal (or under 80%) the item would be considered ‘unresolved’ and worthy of further consideration in future work.

### Patient and public involvement (PPI)

Two PPI representatives (Public Contributors, PCs) were actively involved in all stages of this work. One was a co-applicant on the grant and the other joined us at the start of the project. Their expertise was embedded in all project planning, outputs and future research design. The PCs helped refine the research question and the study protocol and ensured that research design and methods used were appropriate for the study participants, in particular bereaved carers. Their participation also ensured that study documentation (e.g. the participant information sheet, consensus day materials, Delphi and feedback surveys) and project outputs were accessible to all participant groups. The PCs were actively involved in the outcome-mapping process, study management group meetings and both facilitated group discussions on the first consensus day. Reflective log sheets filled both by researchers and PCs were used to monitor and to reflect on how well the steps in the study protocol were achieved and the impact of PPI input into the research.

## Results

### Participant characteristics

Study recruitment lasted from the 3rd of March 2017 to the 13th of April 2018. The sample of the different groups of stakeholders that participated across the different stages of this work are detailed in Table 1

**Table 1:** Participants recruited at each stage of the study

Groups	First Consensus Day	Delphi One	Delphi Two	Second Consensus Day	Feedback Survey
Bereaved People	7	69	30	8	7
Service Providers	11	119	49	8	3
Academic/Researchers	3	33	18	3	1
Members of the Public		19	11	4	
Total	21	240	108	23	11

.

### Outcome extraction and mapping

Results from the outcome mapping exercise are detailed in Table 2, with frequencies given for the descriptor outcome assigned by the research team, and individual frequencies for each outcome as verbatim reported in the quantitative studies used in this exercise. The 105 outcome dimensions defined at this stage are provided in additional file two.

**Table 2:** List of outcomes used in previous studies

Descriptor Outcomes *(Reported verbatim outcomes)*	Overall Frequency
**Anxiety and Depression** *Anxiety (13), Depression (19), Anxiety and Depression (2), Mental Stress (1), Distress (9), Symptom Distress (3), Mental Health (2)*	49
**Grief** *Grief (22), Complicated Grief (2), Blame (1), Despair (1), Knowledge of death and bereavement (1)*	27
**Post Traumatic Stress** A*voidance/intrusion (6), Post-traumatic Stress (3)*	9
**Quality of Life and Wellbeing** *Quality of Life (3), Spiritual Wellbeing (1), Hopelessness (2), Hope (1), Balance (1)*	8
**Coping** *Coping and adaptation (1), coping (2), religious coping (2)*	5
**Self Esteem**	5
**Mood/Affect**	4
**Social Functioning and Adjustment** *Social adjustment (1)* *Social functioning (2)* *Marital strain (1)* *Interpersonal problems* (2)	6
**Physical Health** *Health (1), Physical Health (1), Physical functioning (1)*	3
**Locus of Control**	2
**Interpersonal and Social Support** *Social support (1), Interpersonal relations (1)*	2

### First expert workshop

Two main themes emerged from the group discussions. Participants considered what bereavement services should focus on when supporting service users, and what this might mean in terms of measurable outcomes.

#### Managing and coping with grief

Within all three groups there was talk around the need for bereavement services to enable service users to manage and cope with their grief, rather than try to treat it, and related to this a preference for coping rather than grief to be used as an outcome. Key points included:

- Bereavement support services should aim to support the coping strategies, resilience and “capacity to bear” of service users (bereaved and professional groups).
- Bereavement services should help people to understand the normality of grieving and coping processes and avoid pathologising grief (bereaved and professional groups).
- Service users should be helped to understand the difference between depression and grief, whilst also provided with psychological support to help them process their feelings, reduce feelings of anxiety and panic and improve sleep quality (professional group).
- Bereavement services should help their service users make sense of their experience and loss, enabling them to channel and deal with any feelings of anger that may accompany the loss (e.g. from poor care experiences) (bereaved group), and to “identify maladaptive thoughts and behaviours” (bereaved and professional groups).
- Bereaved people should be supported to remember their loved ones without feeling overwhelmed, and to feel able to enjoy their memories and their sorrow (bereaved and professional groups).

#### Social adjustment, relationships and wellbeing

Other types of identified impacts related to personal and social adjustment and relationships with others, with preferences expressed for support related outcomes. “Improved wellbeing” was identified in one of the professional groups, and a number of different ideas relating to individual, family and social wellbeing were discussed in all three groups. Key points included;

- Services should help bereaved people feel “able to face the future” (bereaved group). This was described as “incremental moves from hopelessness to optimism” in one of the professional groups.
- The re-emergence of sense of self-identity (short and longer term) is important, as is one’s ability to function in life roles and responsibilities, including returning to work and being able to deal with social and financial insecurities (professional groups).
- Bereavement services should help bereaved people manage their often troubled relationships with other family members and close friends (bereaved and professional groups).
- Opportunities for peer support are important. Bereaved participants emphasised the value of talking and being listened to by those with shared experiences, empathy and understanding. Professionals also discussed the need for services to “help with connectedness” and address problems of social isolation (bereaved and professional groups).

Following the discussions that took place on the day, the lists that were originally created from the systematic review were amended to incorporate these discussion points and comments made about the lists. The outcome lists are provided in additional file two, with additions and amendments from the day highlighted.

### Two-round Delphi survey

The outcomes and outcome dimensions identified in stage 1 and 2 of the project were grouped into 17 outcomes and 51 dimensions (additional file two: Lists of outcomes and outcome dimensions). In order to ease the burden on respondents, the dimensions were grouped under the overarching themes of emotional issues, wellbeing, health and support.

A cohort of 240 people took part in the Delphi round 1. Table 3 lists the key characteristics of the respondents.

**Table 3:** Socio-demographic characteristics of participants for Delphi round 1

	All participants	Service users
	N (%)	N (%)
Age		
18 to 24	2 (0.8)	1(1.6)
25 to 34	16 (6.8)	1(1.6)
35 to 44	35 (14.9)	7(11.0)
45 to 54	67 (28.5)	14(21.9)
55 to 64	77 (32.8)	20(31.2)
65 to 74	27 (11.5)	12(18.7)
75 to 79	6 (2.6)	6(9.3)
80 to 84	5 (2.1)	3(4.7)
Gender		
Male	59 (25.1)	17(24.6)
Female	174 (74.0)	51(73.9)
Prefer not to say	1(0.4)	1(1.4)
Ethnicity		
White	217(92.3)	60(93.7)
Mixed	3(1.3)	1(1.5)
Asian or Asian British	6(2.5)	1(1.5)
Black/African/Caribbean/Black British	5(2.1)	2(3.1)
Prefer not to say	2(0.8)	–
Highest qualification		
No qualifications	5(2.1)	5(7.7)
Trade apprenticeship	3(1.3)	2(3.1)
1 or more O level/GCSE’s (at grades A-C)	12(5.1)	7(10.8)
1 or more A levels	10(4.2)	2(3.1)
ONC/OND/ City & Guilds	7(3.0)	2(3.1)
HNC/HND	8(3.4)	3(4.6)
University First Degree (e.g. BA, BSc)	44(18.7)	15(23.1)
Postgraduate Degree (e.g. MA, MSc, PhD)	90(38.2)	16(24.6)
Postgraduate Qualification (e.g. certificate or diploma)	49(20.8)	7(10.8)
Other	7(3.0)	6(9.2)

There was very little missing data, all respondents answered the questions relating to the outcomes and outcomes measures. Four respondents chose not to report age, education and ethnicity, and a further two respondents also did not answer the ethnicity question.

**Fig. 4:**
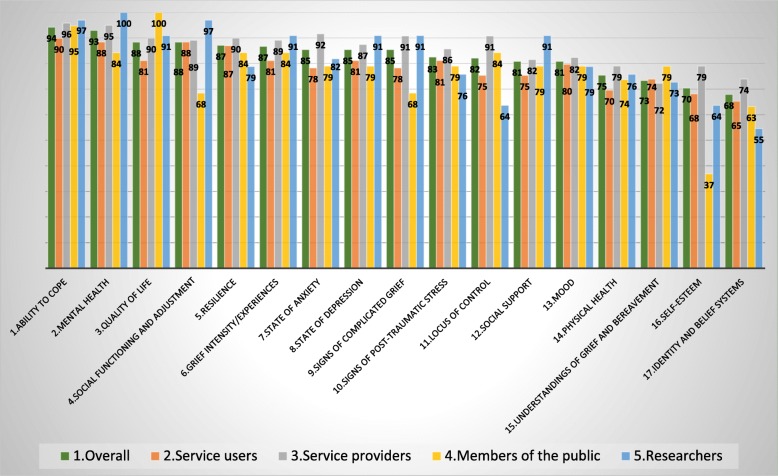
Delphi Round 1: percentages of respondents who thought the outcomes important or very important

The results for the outcomes for the first round of the Delphi is shown in Fig. 4. Against each outcome there is a group of five bars representing the percentage of each stakeholder group who agreed or completely agreed over the importance of the specific outcome being included in the core outcome set and a bar representing the entire cohort score. Two outcomes did not reach the 70% agreement threshold: self-esteem and identity and belief systems. In addition, 17 of the 51 outcome dimensions did not reach the agreement threshold. Hence, these 2 outcomes and 17 outcome dimensions formed the 19 questions included in the Delphi round 2 questionnaire. There was no agreement that any of the outcomes should be considered not important or slightly important (Fig. 4).

Following Delphi round 2, the overall scores for the two outcomes were as follows: ‘self-esteem’ (62%) and ‘identity and belief system’ (62%). It is interesting to note that for most of the outcomes a similar level of agreement was reached across the stakeholder groups in both Delphi rounds.

Table 4 lists the 51 outcome dimensions. For each of them it is indicated if it reached 70% or 80% agreement in the Delphi survey.

**Table 4:** Agreement reached for the outcome dimensions following rounds 1 and 2 of the Delphi survey and consensus day voting

Emotional issues	70%	80%	Shortlisted Consensus Day	Wellbeing	70%	80%	Shortlisted Consensus Day	Health	70%	80%	Shortlisted Consensus Day	Support	70%	80%	Shortlisted Consensus Day
Feelings of loneliness and emptiness	**✓**	**✓**	**✓**	Ability to participate in work^a^	**✓**	**✓**		Anxiety (feelings of tension, nervousness, panic and distress)	**✓**	**✓**	**✓**	Relationships with friends and family	**✓**	**✓**	
Preoccupation with thoughts of the deceased person	**✓**	**✓**		Ability to participate in social or other activities	**✓**	**✓**	**✓**	Depression (a sense of hopelessness, pessimism, periods of crying)	**✓**	**✓**	**✓**	Relationships with health and social care professional(s)	**X**	**X**	
Avoidance of reminders of the deceased person	**X**	**X**		Ability to perform daily tasks	**✓**	**✓**		Related physical symptoms (e.g. pain or sickness)	**✓**			Finding comfort, meaning or strength in religious or spiritual beliefs	**X**	**X**	
Avoidance and denial of distress, grief or other problems	**✓**		**✓**	Involvement in home management and housework	**X**	**X**		Problems with memory, concentration, making decisions, speech	**✓**		**✓**	Accessing practical support if needed	**✓**	**✓**	**✓**
Intensity of grief experienced around time of death	**✓**		**✓**	Financial security and material wellbeing	**✓**			Suicidal thoughts	**✓**	**✓**		Accessing financial/material support if needed^b^	**✓**	**✓**	
Overwhelming thoughts and/or nightmares about loss	**✓**	**✓**		Ability to function as part of a family	**✓**	**✓**		Irritation and bad mood	**✓**			Accessing emotional support if needed	**✓**	**✓**	
Hallucinations about the deceased person	**X**	**X**		Sense of identity and role	**✓**		**✓**	Sleep-related problems	**✓**			Ability to express feelings openly and honestly	**✓**	**✓**	
Feelings of shame and/or stigma	**X**	**X**		Sense of meaning and purpose in life	**✓**	**✓**	**✓**	Tiredness and fatigue^b^	**✓**	**✓**		Accessing guidance if needed	**✓**		
Feelings of detachment and distancing	**✓**			Optimism and hopefulness	**✓**	**✓**		Hyperactivity and inability to slow down^c^	**✓**			Feeling understood by and connected with other bereaved people^b^	**✓**	**✓**	**✓**
Feelings of blame, guilt, anger, bitterness, regret	**✓**	**✓**		Satisfaction with home, neighbourhood and community environment	**X**	**X**		Paranoia or obsessive thoughts^c^	**✓**	**X**					
				Acceptance of grief experiences as normal	**✓**	**✓**	**✓**	Symptoms of phobias	**X**	**X**					
				Acceptance of loss	**✓**	**✓**		Behaviours such as eating disorders or substance abuse	**✓**						
				Understanding and finding meaning of loss	**✓**	**✓**		Self-esteem	**✓**						
				Positive reminiscence and remembering of the deceased^a^	**✓**	**✓**	**✓**	General health problems (e.g. infections, blood pressure, loss of sex drive, other illness)^b^	**✓**	**✓**					
				Regulation and control of feelings and behaviours	**✓**		**✓**	Use of health care services	**X**	**X**	**✓**				
				Ability to find balance and channel grief	**✓**	**✓**	**✓**								
				Ability to take control (e.g. look ahead and start to move forward with life)	**✓**	**✓**	**✓**								

^a^Met 80% threshold in service user sub-group in Delphi 1

^b^Met 80% threshold in service user sub-group in Delphi 2. These items also needed to have been selected on consensus day or final feedback survey to be included

^c^Exceeded 70% in second round of the Delphi survey (service user sub-group)

### Second consensus day results

The second consensus day revolved around a succession of electronic voting sessions. This approach enabled each respondent to independently express their priorities, and as a group, discuss agreement and disagreement with the survey results. Overall, there was substantial agreement between the voting exercise and the Delphi survey. The top six outcomes shortlisted from the voting exercises are listed in Table 5 below and compared with the top six outcomes from the Delphi survey, demonstrating a good level of consistency. The outcome dimensions that were shortlisted from the voting exercises are recorded in Table 4.

**Table 5:** Highest scoring outcomes from Consensus Day and Delphi Survey

Consensus Day Outcomes	Delphi Survey Outcomes
Quality of Life	Ability to Cope
Ability to Cope	Mental Health
Resilience	Quality of life
Social Support	Social functioning and adjustment
Grief intensity/experiences	Resilience
Mental Health	Grief intensity/experiences

### Mapping exercise: outcomes versus dimensions

The mapping exercise that was conducted re-connected the outcome dimensions which exceeded 80% agreement in the Delphi survey with their associated outcomes and was used to help inform and validate the selection of outcomes. This analysis demonstrated that almost all the selected outcome dimensions mapped back to the top three outcomes from the Delphi survey (see additional file three: results of mapping exercise). Whilst there was a clear indication for the first core outcome, ‘Ability to cope’, this exercise also demonstrated significant overlap between the second and third outcomes, ‘Mental health’ and ‘Quality of life’, which converged around the high number of wellbeing related dimensions that were selected. These results thus indicated a need to choose between these two outcomes, and for a wellbeing-oriented definition of ‘Mental health’. Given the grief specific nature of the coping items that were selected, a decision was taken to re-describe ‘ability to cope’ as ‘ability to cope with grief’. This also allowed for several of the grief items that were selected to be accommodated within this outcome.

### Feedback exercise results

The primary purpose of the feedback survey was to establish the preferences of consensus day delegates for the uncertain second core outcome. It also enabled delegates to identify any outcome dimensions proposed for exclusion that they felt should be included in the COS. Eleven delegates responded to the survey, although one only noted their agreement with the study results and did not respond to specific questions. Quality of life was favoured by 6 respondents (4 bereaved people; 1 service provider; 1 researcher), Mental health and wellbeing by 3 respondents (2 bereaved people; 1 service provider) and one respondent described a preference for a combined ‘Quality of life and wellbeing’ outcome. In response to these mixed preferences, the reasoning offered in free text comments, and the apparent convergence around wellbeing related dimensions a decision was made to describe the second outcome as ‘Quality of life and mental wellbeing’.

No new outcome dimensions were included following the feedback survey results, although five were considered ‘unresolved’ according to our stated criteria (50 to 79% in favour of inclusion). These five items were: Sense of identity and role; Problems with memory, concentration, making decisions, speech; Accessing financial/material support if needed; Tiredness and Fatigue; General health problems (eg infections, blood pressure). These items may warrant further consideration in future work in this area.

### Core outcomes and dimensions

The selected outcomes and dimensions are presented below. For ease of interpretation the outcome dimensions have been categorised under 9 thematic headings (Table 6).

**Table 6:** Core Outcomes with Dimensions

Ability to Cope with Grief	Quality of Life and Mental Wellbeing
**Negative and overwhelming grief** • Feelings of loneliness and emptiness • Feelings of blame, guilt, anger, bitterness, regret • Overwhelming thoughts and/or nightmares about loss • Preoccupation with thoughts of the deceased	**Participation in work and/or other regular activities** • Ability to perform daily tasks • Ability to participate in work • Ability to participate in social activities
**Communication and connectedness** • Ability to express feelings openly and honestly • Feeling understood by and connected with other bereaved people	**Relationships and social functioning** • Ability to function as part of a family • Relationships with friends and family
**Understanding, accepting and finding meaning in grief** • Acceptance of grief experiences as normal • Understanding, acceptance, finding meaning in loss • Positive reminiscence and remembering of the deceased	**Positive mental wellbeing** • Sense of meaning and purpose in life • Optimism and hopefulness
**Finding balance between grief and life going forwards** • Ability to find balance and channel grief • Ability to take control/ look ahead and start to move forward with life	**Negative mental & emotional state** • Anxiety (feelings of tension, nervousness, panic and distress) • Depression (a sense of hopelessness, pessimism, periods of crying) • Suicidal thoughts
**Accessing appropriate support** • Accessing emotional support if needed • Accessing practical support if needed	

## Discussion

Through an extensive process of outcome identification, mapping and stakeholder consultations this piece of work has identified two core outcomes and associated outcome dimensions that can be used to inform the design and evaluation of adult bereavement support in palliative care. The stakeholder-based selection of two outcomes, ‘Ability to cope with grief’ and ‘Quality of life and mental wellbeing’ offers a consistent way forward for researchers and practitioners working in the field. It also represents a different conceptual approach for evaluating bereavement interventions than the medicalised and pathological approaches dominant in the published literature, aligning more closely with public health and resilience-based approaches to bereavement care [12, 13]. The implications of these results for future evaluation, research and service design are discussed.

The need for a set of core outcomes that can be used in evaluations of adult bereavement support interventions was confirmed in the outcome mapping exercise conducted at the start of this study. The identification of 34 differently described outcomes and many more measurement instruments supports previous observations of problematic and inconsistent outcome measurement in bereavement research [4, 14, 15, 17–19]. The stakeholder-based identification of two core outcomes, ‘Ability to cope with grief’ and ‘Quality of life and mental wellbeing’ is a first important step towards facilitating a more consistent and useful approach to outcome measurement in this field of research and evaluation. In doing so it also complements sets of consensus-based service standards that have been recently developed [11, 21], or are in current development (https://www.eapcnet.eu/eapc-groups/task-forces/bereavement).

This selection of outcomes appears to favour an alternative conceptual approach for evaluating bereavement care to the more pathological approaches which have dominated in the published research literature. They instead align more closely with public health and resilience-based approaches which emphasise the importance of social networks and an appropriate mix of community based and specialist support [12, 13]. The need for a shift towards coping, support and wellbeing outcomes was explicitly articulated by participants at our stakeholder workshops. The apparent divergence between the outcomes most commonly used by researchers in the published literature (Grief, Depression, Anxiety) and the outcomes which were most popular in our consensus days and Delphi surveys (Coping, Wellbeing, Quality of Life, Mental Health, Social Support) also further confirms this. Although aspects of grief experience, depression and anxiety are represented in the two outcomes, the overall orientation of the outcomes is more positive, addressing both individual and social dimensions of coping, resilience and wellbeing during bereavement. As a COS there is an assumption that improvements in these areas would occur for bereaved individuals accessing an ‘effective’ program of bereavement support. Whilst improvements in some dimensions may more likely represent direct impacts of the support (e.g. in ‘Communication and connectedness’), improvements in others may be more likely to occur as indirect impacts, indicative of generalised improvements to wellbeing (e.g. ‘Participation in work and/or regular activities’).

There are several factors that may explain this apparent departure from the more disease focused outcomes used in previous studies. One reason may be the relatively high number of Randomised Control Trials (RCTs) of grief therapy interventions that were included in the mapping exercises and a possible preference amongst these researchers for pathological outcomes. The majority of participants in our consensus days and Delphi survey were service providers and service users, with researcher participants also representing a diversity of methodological backgrounds. The scope of our COS was not restricted to grief therapy, and instead intended to cover the wide-ranging types of support provided in palliative care settings, to meet the varying needs of service users. The consensus exercises therefore represented the perspectives of a much wider range of stakeholders, than those who design and test grief therapy interventions, further reinforcing the importance of wide-ranging stakeholder participation when developing COS.

Following this, it is also worth noting that in the Delphi results, voting exercises and expert discussions, many of the preferences and priorities to emerge were for outcomes and outcome dimensions that either originated from the qualitative literature and/or group discussions, or were only marginally represented in the quantitative measures used in previous evaluations. This would suggest that there may not just be a problem with consistency but also the appropriateness of some of the outcomes commonly used in research studies. This re-ignites the question of whether the inconclusiveness and lack of positive results reported in many bereavement trials and reviews (e.g. 4, 14–16), might in part be explained by the choice of outcomes and measures, rather than poor efficacy of the intervention or service [15]. Whilst a recent mixed-methods review of bereavement support in palliative care found limited evidence of positive effects in the RCT studies, there were a number of positive impacts consistently identified in the qualitative and mixed methods evaluations that were included in the review. Several of these are now represented in the outcome set reported here e.g. ‘facilitating loss and grief resolution’, ‘restoration and moving on’, ‘acquisition of coping strategies’ and ‘social support’ [14]. This again reiterates the importance of using existing qualitative work, combined with stakeholder discussions, for identifying and defining potential outcomes that may otherwise get missed out of evaluation study protocols.

### Limitations, strengths and implications for further research

One difficulty with this piece of work concerned the potential variation in how survey items are interpreted and understood by participants. Steps taken to mitigate the effects of participant subjectivity included open discussions in consensus days, extensive piloting of the survey, and active PPI involvement throughout. The high degree of consistency between the results of both consensus days and the Delphi Survey, and between the outcomes and dimensions that were prioritised, also helps to validate these results. The relatively high numbers of bereaved and service user participants are another strength of this work, helping to ensure that our outcome sets represent the views and experiences of those who matter most when designing and evaluating services. However, there was inevitably some self-selection bias and lower socio-economic status and minority ethnic groups were under-represented in our sample.

The next step for this ongoing piece of work is to identify, adapt and develop tools which specifically address these two outcomes. A preliminary ‘best fit’ analysis of existing validated measures used during the initial outcome mapping process found no tools that completely covered these dimensions but identified some with apparent potential to be combined or adapted to cover the outcomes. Those with ‘best fit’ included grief specific measures connected with coping, resilience and meaning based frameworks, such as the Inventory for Daily Widowed Life [26], Adult Attitude to Grief Scale [27], Grief and Meaning Reconstruction Inventory [28]. There were also some generic quality of life and wellbeing tools which demonstrated good coverage of the selected quality of life and wellbeing dimensions e.g. Multicultural Quality of Life Index, [29] Warwick-Edinburgh Mental Wellbeing Scale [30]. Updated searches to identify any new measures and rigorous content analysis, quality appraisal and stakeholder consultation for all potentially relevant measures is needed following the COMET and COSMIN guidance [31, 32]. This will enable robust recommendations for any existing outcome measures considered suitable for this COS and the identification of any additional validation or development work still required. Given the acknowledged difficulties of conducting trials in complex interventions generally [33], and in palliative care and bereavement specifically [34, 35], consideration is also being given to how tools could be developed to be useful for other types of evaluation designs, to serve the needs of clinical practice and research [36].

## Conclusion

This project has identified two core outcomes and associated dimensions which can inform the design and evaluation of adult bereavement support in palliative care. The stakeholder based selection of two outcomes, ‘Ability to cope with grief’ and ‘Quality of life and mental wellbeing’ begins to pave a more consistent way forward for researchers and practitioners working in the field, as well as a departure from the more medicalised approaches and pathological outcomes typically seen in the published quantitative literature. Further work is being planned to identify and develop measures which are specific to this core outcome set, and which will facilitate the future comparability of services or interventions, from both clinical and research perspectives.

## Supplementary information

**Additional file 1.** List of all measures used to construct Delphi survey lists.

**Additional file 2.** Integrated lists of outcomes and outcome dimensions developed from systematic review and consensus day discussions.

**Additional file 3.** Results of outcomes/dimensions mapping exercise.

## Data Availability

The datasets generated and analysed during the current study are available in a separate report in the Cardiff University repository, http://orca.cf.ac.uk/128233/

## Abbreviations

COS: Core Outcome Set
EoL: End of Life
NICE: National Institute for Clinical Excellence
PC: Public Contributors
PPI: Patient and Public Involvement
RCT: Randomised Control Trial
