# Supplementary material for: Coping and wellbeing in bereavement: two core outcomes for evaluating bereavement support in palliative care
Source: BMC Palliat Care. 2020 Mar 12;19:29. doi: 10.1186/s12904-020-0532-4 (PMC7068975; doi:10.1186/s12904-020-0532-4)
Supplement: Supplementary file 1 — Additional file 1. List of all measures used to construct Delphi survey lists. [file 12904_2020_532_MOESM1_ESM.docx]

**Additional File One-List of all measures used to construct Delphi Survey lists**

| **Grief Measures**  *Adult Attitude to Grief Scale*  Sim J, Machin L, Bartlam B. Identifying vulnerability in grief: psychometric properties of the Adult Attitude to Grief Scale. Quality of Life Research. 2014 May 1;23(4):1211-20.  *Bereavement Risk Index*  Parkes CM. Bereavement. In: Doyle D, Hanks GCW, MacDonald N (eds), Oxford Textbook of Palliative Medicine. Oxford: Oxford University Press, 1993, p.663–678.  *Core Bereavement Items*  Burnett P, Middleton W, Raphael B, Martinek N. Measuring core bereavement phenomena. Psychol Med 1997; 27: 49–57.  *Grief and Meaning Reconstruction Inventory*  Gillies JM, Neimeyer RA, Milman E. The grief and meaning reconstruction inventory (GMRI): Initial validation of a new measure. Death Studies. 2015 Feb 7;39(2):61-74.  *Grief Experience Questionnaire*  Barrett TW, Scott TB. Development of the grief experience questionnaire. Suicide and Life‐Threatening Behavior. 1989 Jun;19(2):201-15.  *Grief Evaluation Measure*  Jordan JR, Baker J, Matteis M, Rosenthal S, Ware ES. The Grief Evaluation Measure (GEM): an initial validation study. Death Stud 2005; 29: 301–332  *Grief Reaction Index*  Lennon MC, Martin JL, Dean L. The influence of social support on AIDS-related grief reaction among gay men. Social Science & Medicine. 1990 Jan 1;31(4):477-84.  *Grief Recovery Questions*  Lehman, D., Wortman, C., & Williams, A. (1987). Long‐term effects of losing a spouse or child in a motor vehicle crash. Journal of Personality and Social Psychology, 52, 218–231.  Lehman, D., Ellard, J., & Wortman, C. (1986). Social support for the bereaved: Recipients' and providers' perspectives in what is helpful. Journal of Consulting and Clinical Psychology, 54, 438–446.  *Hogan Grief Reaction Checklist*  S. Hogan, Daryl B. Greenfield, Lee A. Schmidt N. Development and validation of the Hogan grief reaction checklist. Death studies. 2001 Jan 1;25(1):1-32.  *Integration of Stressful Life Experiences Scale*  Holland JM, Currier JM, Coleman RA, Neimeyer RA. The Integration of Stressful Life Experiences Scale (ISLES): Development and initial validation of a new measure. International Journal of Stress Management. 2010 Nov;17(4):325.  *Inventory of Complicated Grief*  Prigerson HG, Maciejewski PK, Reynolds III CF, Bierhals AJ, Newsom JT, Fasiczka A, Frank E, Doman J, Miller M. Inventory of Complicated Grief: a scale to measure maladaptive symptoms of loss. Psychiatry research. 1995 Nov 29;59(1-2):65-79.  *Inventory of Complicated Spiritual Grief*  Burke LA, Neimeyer RA, Holland JM, Dennard S, Oliver L, Shear MK. Inventory of Complicated Spiritual Grief: Development and validation of a new measure. Death studies. 2014 Apr 21;38(4):239-50.  *Inventory of Daily Widowed Life*  Caserta MS, Lund DA. Toward the development of an Inventory of Daily Widowed Life (IDWL): Guided by the dual process model of coping with bereavement. Death Studies. 2007 Jun 11;31(6):505-35.  *Inventory of Traumatic Grief;*  Prigerson H, Kasl S, Jacobs S. Inventory of traumatic grief. Handbook of bereavement research: Causes, consequences and care. 2001:638-45.  *Meaning of Loss Codebook*  Gillies J, Neimeyer RA, Milman E. The meaning of loss codebook: Construction of a system for analyzing meanings made in bereavement. Death Studies. 2014 Apr 21;38(4):207-16.  *Perceived Life Significance Scale*  Hibberd R, Vandenberg B. Development and validation of the perceived life significance scale. Death studies. 2015 Jul 3;39(6):369-83.  *Texas Revised Inventory of Grief*  Faschingbauer TR. Texas revised inventory of grief. 1981.  *Two Track Bereavement Questionnaire for Complicated Grief*  Rubin SS, Bar-Nadav O. The Two-Track Bereavement Questionnaire for complicated grief (TTBQ-CG31). Techniques of Grief Therapy: Assessment and Intervention. 2015 Sep 25:87. |
| --- |
| **Anxiety/Depression/Distress Measures**  *Beck Anxiety Inventory*  Beck AT, Epstein N, Brown G, Steer RA. An inventory for measuring clinical anxiety: psychometric properties. Journal of consulting and clinical psychology. 1988 Dec;56(6):893.  *Beck Depression Inventory*  Beck AT, Steer RA, Brown GK. Beck depression inventory-II. San Antonio. 1996;78(2):490-8.  *Brief Symptom Inventory*  Derogatis LR, Melisaratos N. The brief symptom inventory: an introductory report. Psychological medicine. 1983 Aug;13(3):595-605.  *Centre for Epidemiologic Studies Depression Scale*  Irwin M, Artin KH, Oxman MN. Screening for depression in the older adult: criterion validity of the 10-item Center for Epidemiological Studies Depression Scale (CES-D). Archives of internal medicine. 1999 Aug 9;159(15):1701-4.  *Depression Anxiety Stress Scales*  Crawford JR, Henry JD. The Depression Anxiety Stress Scales (DASS): Normative data and latent structure in a large non‐clinical sample. British journal of clinical psychology. 2003 Jun;42(2):111-31.  *General Health Questionnaire*  Banks MH, Clegg CW, Jackson PR, Kemp NJ, Stafford EM, Wall TD. The use of the General Health Questionnaire as an indicator of mental health in occupational studies. Journal of occupational psychology. 1980 Sep;53(3):187-94.  *Geriatric Depression Scale*  Yesavage JA, Brink TL, Rose TL, Lum O, Huang V, Adey M, Leirer VO. Development and validation of a geriatric depression screening scale: a preliminary report. Journal of psychiatric research. 1982 Jan 1;17(1):37-49.  *Hamilton Rating Scale (anxiety)*  Hamilton MA. The assessment of anxiety states by rating. British journal of medical psychology. 1959 Mar 1;32(1):50-5.  *Hamilton Rating Scale (depression)*  Hamilton M. The Hamilton rating scale for depression. InAssessment of depression 1986 (pp. 143-152). Springer, Berlin, Heidelberg.  *Hopkins Symptom Checklist 25*  Derogatis LR, Lipman RS, Rickels K, Uhlenluth EH, Covi L (1974) The Hopkins Symptom Checklist (HSCL): a self report symptom inventory. Behav Sci 19: 1±15  Hospital Anxiety & Depression Scale;  Zigmond AS, Snaith RP. The hospital anxiety and depression scale. Acta psychiatrica scandinavica. 1983 Jun 1;67(6):361-70.  Self-rating Depression Scale;  Zung WW. A self-rating depression scale. Archives of general psychiatry. 1965 Jan 1;12(1):63-70.  State Trait Anxiety Inventory;  Spielberger CD. Manual for the State-Trait Anxiety Inventory STAI (form Y)(" self-evaluation questionnaire").  Subjective Units of Distress Scale;  Wolpe J, Lazarus AA. Behavior therapy techniques: A guide to the treatment of neuroses.  Symptom Checklist 90.  Derogatis LR, Cleary PA. Confirmation of the dimensional structure of the SCL‐90: A study in construct validation. Journal of clinical psychology. 1977 Oct;33(4):981-9. |
| **Mood Measures**  Multiple Affect Adjective Checklist;  Lubin B, Van Whitlock R, Reddy D, Petren S. A comparison of the short and long forms of the Multiple Affect Adjective Check List—Revised (MAACL‐R). Journal of clinical psychology. 2001 Mar;57(3):411-6.  Positive and Negative Affect Schedule;  Watson D, Clark LA, Tellegen A. Development and validation of brief measures of positive and negative affect: the PANAS scales. Journal of personality and social psychology. 1988 Jun;54(6):1063.  Profile of Mood States;  Curran SL, Andrykowski MA, Studts JL. Short form of the profile of mood states (POMS-SF): psychometric information. Psychological assessment. 1995 Mar;7(1):80. |
| **Post Traumatic Stress Measures**  Impact of Event Scale;  Horowitz M, Wilner N, Alvarez W. Impact of Event Scale: A measure of subjective stress. Psychosomatic medicine. 1979 May 1;41(3):209-18.  PTSD Checklist Scale;  Blanchard EB, Jones-Alexander J, Buckley TC, Forneris CA. Psychometric properties of the PTSD Checklist (PCL). Behaviour research and therapy. 1996 Aug 1;34(8):669-73.  Structured Clinical Interview for DSM IV  Gibbon M, Spitzer RL, Williams JB, Benjamin LS, First MB. Structured clinical interview for DSM-IV axis II personality disorders (SCID-II). Am Psych Pub. 1997. |
| **Self Esteem Measures**  Self Esteem Scale  Rosenberg M. Rosenberg self-esteem scale (SES). Society and the adolescent self-image. 1965. |
| **Social Support Measures**  Arizona Social Support Interview Schedule;  Barrera M. Arizona social support interview schedule. Social Networks and Social Support. 1988:88-93.  ENRICHD Social Support Inventory  Mitchell PH, Powell L, Blumenthal J, Norten J, Ironson G, Pitula CR, Froelicher ES, Czajkowski S, Youngblood M, Huber M, Berkman LF. A short social support measure for patients recovering from myocardial infarction: the ENRICHD Social Support Inventory. Journal of Cardiopulmonary Rehabilitation and Prevention. 2003 Nov 1;23(6):398-403.  Inventory of Social Support  Hogan NS, Schmidt LA. Inventory of Social Support (ISS). Techniques of grief therapy: Assessment and intervention. 2016:99-103.  Social Provisions Scale  Gottlieb BH, Bergen AE. Social support concepts and measures. Journal of psychosomatic research. 2010 Nov 1;69(5):511-20.  Social Support Questionnaire  Gottlieb BH, Bergen AE. Social support concepts and measures. Journal of psychosomatic research. 2010 Nov 1;69(5):511-20. |
| **Social adjustment Measures**  Inventory of Interpersonal Problems  Horowitz LM, Rosenberg SE, Baer BA, Ureño G, Villaseñor VS. Inventory of interpersonal problems: psychometric properties and clinical applications. Journal of consulting and clinical psychology. 1988 Dec;56(6):885.  Social Adjustment Scale - Self-Report;  Weissman MM, Bothwell S. Assessment of social adjustment by patient self-report. Archives of general psychiatry. 1976 Sep 1;33(9):1111-5.  Work and Social Adjustment Scale  Mundt JC, Marks IM, Shear MK, Greist JM. The Work and Social Adjustment Scale: a simple measure of impairment in functioning. The British Journal of Psychiatry. 2002 May;180(5):461-4. |
| **Coping Measures**  Brief COPE Inventory;  Carver CS. You want to measure coping but your protocol’too long: Consider the brief cope. International journal of behavioral medicine. 1997 Mar 1;4(1):92.  Religious Coping RCOPE;  Pargament KI, Koenig HG, Perez LM. The many methods of religious coping: Development and initial validation of the RCOPE. Journal of clinical psychology. 2000 Apr;56(4):519-43.  Ways of Coping Checklist;  Scherer RF, Luther DC, Wiebe FA, Adams JS. Dimensionality of coping: Factor stability using the ways of coping questionnaire. Psychological Reports. 1988 Jun;62(3):763-70.  Coping Styles Questionnaire 2010;  Roger D, Jarvis G, Najarian B. Detachment and coping: The construction and validation of a new scale for measuring coping strategies. Personality and Individual differences. 1993 Dec 1;15(6):619-26.  Coping Resources Inventory;    Matheny KB, Aycock DW, Curlette WL, Junker GN. The coping resources inventory for stress: A measure of perceived resourcefulness. Journal of Clinical psychology. 1993 Nov;49(6):815-30.  The Hannover Coping Manual;  Geyer S, Ellis R, Koch-Gießelmann H (2009) Das Hannoversche Copinginventar: Ein qualitatives Verfahren zur Untersuchung der Problembewältigung bei Frauen mit Mammakarzinom. In: Neises M, Weidner K (eds) Qualitative Forschungsansätze und Ergebnisse in der psychosomatischen Frauenheilkunde, 1st edn. Pabst Science Publisher, Lengerich, pp 173–193  Coping Response Inventory;  Moos RH. Coping Responses Inventory: CRI-Adult Form, Professional Manual. 1993. Florida: Psychological Assessment Resources. |
| **Quality of Life Measures**  Assessment of Quality of Life Instrument  Hawthorne G, Richardson J, Osborne R. The Assessment of Quality of Life (AQoL) instrument: a psychometric measure of health-related quality of life. Quality of Life Research. 1999 May 1;8(3):209-24.  Euro QoL 5 dimensions questionnaire (EQ5D);  <https://euroqol.org/eq-5d-instruments/>  Functional Assessment of HIV Infection;  Cella DF, McCain NL, Peterman AH, Mo F, Wolen D. Development and validation of the Functional Assessment of Human Immunodeficiency Virus Infection (FAHI) quality of life instrument. Quality of Life Research. 1996 Aug 1;5(4):450-63.  International Quality of Life Assessment Short Form-36;  Ware Jr JE, Gandek B. Overview of the SF-36 health survey and the international quality of life assessment (IQOLA) project. Journal of clinical epidemiology. 1998 Nov 1;51(11):903-12.  Multicultural Quality of Life Index;  Mezzich, J. E., Cohen, N. L., Ruiperez, M. A., Banzato, C. E. and Zapata‐Vega, M. I. (2011), The Multicultural Quality of Life Index: presentation and validation. Journal of Evaluation in Clinical Practice, 17: 357-364. doi:[10.1111/j.1365-2753.2010.01609.x](https://doi.org/10.1111/j.1365-2753.2010.01609.x)  Nottingham Health Profile;  Hunt, S. M., McKenna, S. P., McEwen, J., Williams, J., & Papp, E. (1981). The Nottingham health profile: Subjective health status and medical consultations. Social Science & Medicine. Part A: Medical Psychology & Medical Sociology, 15(3, Part 1), 221–229.  Quality of Life Inventory;  Frisch MB, Cornell J, Villanueva M, Retzlaff PJ. Clinical validation of the Quality of Life Inventory. A measure of life satisfaction for use in treatment planning and outcome assessment. Psychological assessment. 1992 Mar;4(1):92.  Quality of Well-being Scale  Kaplan RM, Ganiats TG, Sieber WJ, Anderson JP. The Quality of Well-Being Scale: critical similarities and differences with SF-36. International Journal for Quality in Health Care. 1998 Dec 1;10(6):509-20.  ReQoL (Recovering QoL);  Keetharuth AD, Brazier J, Connell J, Bjorner JB, Carlton J, Buck ET, Ricketts T, McKendrick K, Browne J, Croudace T, Barkham M. Recovering Quality of Life (ReQoL): a new generic self-reported outcome measure for use with people experiencing mental health difficulties. The British Journal of Psychiatry. 2018 Jan;212(1):42-9.  World Health Organisation Quality of Life Instrument  Group TW. The World Health Organization quality of life assessment (WHOQOL): development and general psychometric properties. Social science & medicine. 1998 Jun 15;46(12):1569-85. |
| **Health Measures**  Health Status/Health Behaviors Scale (HHB);  A. MURPHY CLARK JOHNSON KEVIN C. CAIN ABHIJIT DAS GUPTA MARGARET DIMOND JANET LOHAN ROBERT BAUGHER SH. Broad-spectrum group treatment for parents bereaved by the violent deaths of their 12-to 28-year-old children: A randomized controlled trial. Death Studies. 1998 Mar 1;22(3):209-35.  SF-12 Health Survey  Ware Jr JE, Kosinski M, Keller SD. A 12-Item Short-Form Health Survey: construction of scales and preliminary tests of reliability and validity. Medical care. 1996 Mar 1:220-33. |
| **Mental Health/Wellbeing Measures**  GHQ;  Goldberg DP, Hillier VF. A scaled version of the General Health Questionnaire. Psychological Medicine. Cambridge University Press; 1979;9(1):139–45.  ICECAP-A (ICEpop CAPability measure for Adults);  Al-Janabi H, Flynn TN, Coast J. Development of a self-report measure of capability wellbeing for adults: the ICECAP-A. Quality of Life Research. 2012 Feb 1;21(1):167-76.  Life Orientation Test - Revised;  Segerstrom SC, Evans DR, Eisenlohr-Moul TA. Optimism and pessimism dimensions in the Life Orientation Test-Revised: Method and meaning. Journal of Research in Personality. 2011 Feb 1;45(1):126-9.  Warwick-Edinburgh Mental Well-being Scale  Tennant R, Hiller L, Fishwick R, Platt S, Joseph S, Weich S, Parkinson J, Secker J, Stewart-Brown S. The Warwick-Edinburgh mental well-being scale (WEMWBS): development and UK validation. Health and Quality of life Outcomes. 2007 Dec;5(1):63  Functional Assessment Chronic Illness Therapy–Spiritual (FACIT–Sp)  Bredle JM, Salsman JM, Debb SM, Arnold BJ, Cella D. Spiritual well-being as a component of health-related quality of life: the functional assessment of chronic illness therapy—spiritual well-being scale (FACIT-Sp). Religions. 2011 Mar;2(1):77-94.  Geriatric Hopelessness Scales;  Fry PS. Development of a geriatric scale of hopelessness: Implications for counseling and intervention with the depressed elderly. Journal of Counseling Psychology. 1984 Jul;31(3):322.  Herth Hope Index;  Herth K. Abbreviated instrument to measure hope: development and psychometric evaluation. Journal of advanced nursing. 1992 Oct;17(10):1251-9. |
| **Locus of Control Measures**  Rotter's forced-choice Internal-External Locus of Control Scale;  Rotter, Julian B (1966). "Generalized expectancies for internal versus external control of reinforcement". Psychological Monographs: General and Applied. **80**: 1–28. [*doi*](https://en.wikipedia.org/wiki/Digital_object_identifier):[*10.1037/h0092976*](https://doi.org/10.1037%2Fh0092976).  Ziegler-Reid State Locus of Control Measure;  Reid DW, Zeigler M. The desired control measure and adjustment among the elderly In Lefcourt H, editor.,eds 1981. Research with the Locus of Control Construct. |
| **Resilience**  Baruth Protective Factors Inventory;  Baruth KE, Caroll JJ. A formal assessment of resilience: The Baruth Protective Factors Inventory. The Journal of Individual Psychology. 2002.  Brief Resilience Scale;  Smith BW, Dalen J, Wiggins K, Tooley E, Christopher P, Bernard J. The brief resilience scale: assessing the ability to bounce back. International journal of behavioral medicine. 2008 Sep 1;15(3):194-200.  Brief Resilient Coping Scale;  Sinclair VG, Wallston KA. The development and psychometric evaluation of the Brief Resilient Coping Scale. Assessment. 2004 Mar;11(1):94-101.  Connor-Davidson Resilience Scale;  Connor KM, Davidson JR. Development of a new resilience scale: The Connor‐Davidson resilience scale (CD‐RISC). Depression and anxiety. 2003 Sep;18(2):76-82.  Dispositional Resilience Scale;  Bartone PT. Test-retest reliability of the dispositional resilience scale-15, a brief hardiness scale. Psychological reports. 2007 Dec;101(3):943-4.  Resilience Scale;  Wagnild GM, Young HM: Development and psychometric evaluation of the resilience scale. Journal of Nursing Measurement 1993,1(2):165–178.    Resilience Scale for Adults;  Friborg O, Hjemdal O, Rosenvinge JH, Martinussen M. A new rating scale for adult resilience: what are the central protective resources behind healthy adjustment?. International journal of methods in psychiatric research. 2003 Jun;12(2):65-76. |
