# Supplementary material for: Coping and wellbeing in bereavement: two core outcomes for evaluating bereavement support in palliative care
Source: BMC Palliat Care. 2020 Mar 12;19:29. doi: 10.1186/s12904-020-0532-4 (PMC7068975; doi:10.1186/s12904-020-0532-4)
Supplement: Supplementary file 2 — Additional file 2. Integrated lists of outcomes and outcome dimensions developed from systematic review and consensus day discussions. [file 12904_2020_532_MOESM2_ESM.docx]

**Additional File Two: Integrated lists of outcomes and outcome dimensions developed from systematic review and consensus day discussions**

Key:

Underlined = confirmed by qualitative studies in systematic review

Dimension*=added from list of qualitative themes

Coloured text added following discussions at expert workshop; Bereaved group; Professional group 1; Professional group2

**Grief**

| **Grief Dimensions** |
| --- |
| 1. Physical health problems e.g. running nose, chest pains, dizziness, palpitations |
| 1. Anxiety, worry and panic behaviour |
| 1. Self-destructive behaviour |
| 1. Cognitive reactions such as difficulty concentrating, remembering. |
| 1. Sadness and crying |
| 1. Hopelessness, pessimism, loss of meaning and purpose |
| 1. Loneliness and emptiness |
| 1. Painful, intrusive thoughts (e.g. memories of suffering and death*) |
| 1. Preoccupation with thoughts of deceased. |
| 1. Feelings of blame, guilt, anger, bitterness, regret (e.g. over care/ death experiences of loved one, family conflict*) |
| 1. Shame and stigmatisation |
| 1. Detachment and disconnection from self and others |
| 1. Seeking an understanding for why death occurred |
| 1. Non acceptance of death/disbelief |
| 1. Avoidance and denial of distress and grief |
| 1. Avoidance and denial of thoughts, feelings, reminders of deceased |
| 1. Reminiscence (and maintaining emotional closeness*) (having time to think about the person and enjoy memories, yearning) |
| 1. Coping ability and resilience (both discovering resilience and strengthening it) |
| 1. Support/ lack of support from family/friends |
| 1. Personal growth, new roles and responsibilities, optimism/hopefulness |
| 1. Loss of role and identity* |
| 1. Intensity of grief around the time of the death |
| 1. Auditory and visual hallucinations |
| 1. Pain in same parts of body as experienced by deceased |
| 1. Dreaming of the deceased (positive experience) |

Psychological outcomes

| **Anxiety and depression** |
| --- |
| 1. General state of anxiety, characterised by feelings of tension, nervousness, panic and distress. /Reduced anxiety/ reduced panic |
| 1. Lack of motivation and loss of interest or enjoyment in one’s job, leisure activities and social life. |
| 1. General state of depressed mood, characterised by a sense of hopelessness, pessimism and periods of crying. |
| 1. Wide range of physical or physiological symptoms as a result of depression or anxiety. |
| 1. Cognitive symptoms e.g. problems with memory and concentration, decision making |
| 1. Feelings of irritation and annoyance |
| 1. Feelings of self-blame and guilt |
| 1. Suicidal thinking |
| 1. Sleep-related problems, including insomnia. / improved sleep quality |
| 1. Hyperactivity, inability to slow down (e.g. engagement in displacement activities) |
| 1. Excess tiredness |
| 1. Slowing down of movement, speech and thoughts. |
| 1. Impact on libido |
| 1. Anxiety or distress as a result of perceived presence of physical symptoms (eg pain) or worry about having a serious illness |
| 1. Symptoms relating to paranoia, obsessive thoughts, feelings of discrimination |
| 1. Symptoms relating to phobias |
| 1. General state of calmness |
| 1. Capability for insight |

| **Mood** |
| --- |
| 1. Feelings of anxiety, depression and distress |
| 1. Fatigue |
| 1. Anger and hostility |
| 1. Tension |
| 1. Loss of vigour |
| 1. Feeling positive e.g. enthusiastic, alert, active |
| 1. Sensation seeking |

| **Post-traumatic stress** |
| --- |
| 1. Avoiding activities and feeling distant (also common grief symptoms) |
| 1. Arousal e.g. difficulty falling asleep, concentrating, easily startled (also common grief symptoms) |
| 1. Intrusive thoughts and nightmares (also common grief symptoms) |
| 1. Eating and substance abuse disorders |
| 1. Bodily symptoms caused by mental illness e.g. pain |
| 1. Psychotic symptoms and disorders |
| 1. Mood episodes and disorders |

| **Self-esteem** |
| --- |
| 1. Feelings about yourself |

Cognitive outcomes

| **Cognitive dimensions** |
| --- |
| 1. Meaning making |
| 1. Understanding normality of grief and consequences for others |
| 1. Understanding others’ behaviours and actions |
| 1. Identifying maladaptive thoughts and behaviours |

Physical health

| **Physical health** |
| --- |
| 1. Health status and problems e.g. infections, blood pressure, other illnesses |
| 1. Health behaviours |
| 1. Health care use (e.g. visits to GP or primary care as a result of bereavement) |
| 1. Mortality rates |

Coping, adaptation and quality of life

| **Social functioning and adjustment** |
| --- |
| 1. Ability to work (or return to work following bereavement) |
| 1. Ability to perform home management and housework tasks (including hoarding behaviour) |
| 1. Participation in social activities |
| 1. Participation in private leisure activities or hobbies |
| 1. Relationships with family |
| 1. Relationship with spouse |
| 1. Relationships with others (outside of family) and with community/ Relationships and communication |
| 1. Ability to function in life roles and responsibilities |
| 1. Forming new roles and relationships* (adapting to change post-carer role) |
| 1. Forming new routines and structure to the day* (sense of normality) |
| 1. Functioning as a parent and/or in family unit (being bereaved with additional caring responsibilities) |
| 1. Managing conflict or misunderstanding (of different ways of dealing with grief)/ Managing how others treat them |
| 1. Personality and behavioural traits e.g. assertive, controlling, submissive |

| **Coping and resilience** |
| --- |
| 1. Efforts to detach and distance oneself from a stressful situation |
| 1. Efforts or ability to control one’s feelings and behaviours (e.g. to channel anger) |
| 1. Efforts or ability to find balance and channel grief i.e. focus on grief at certain times and focus on other areas of life at other times.* (Being able to ‘enjoy’ sorrow, ‘pure’ grief) |
| 1. Ability to live with the grief and function in the external world (emotional and self resilience)/ minimizing negative consequences of grief/help with psychological wellbeing & capacity to bear/ coping with feelings of loss and grief, discovering and strengthening resilience |
| 1. Efforts or ability to self-manage and rely less on health services |
| 1. Efforts or ability to access support and maintain relationships |
| 1. Efforts to escape or avoid problems |
| 1. Efforts or ability to accept responsibility, take control and alter the situation/address the problem (e.g. look ahead and move forwards with life*)/ Being |
| 1. Efforts or ability to think positively, find meaning and hope in new life situation and/ or the future (incremental moves from hopelessness to optimism) |
| 1. Efforts or ability to accept, understand and find meaning in loss |
| 1. Efforts or ability to accept and view grief experiences as normal* / understanding that experiences are normal/ understanding normality of grieving process/understanding difference between grief and depression/not pathologising or medicalising grief |
| 1. Efforts or ability to find comfort, meaning or strength in religious or spiritual beliefs* |
| 1. Efforts or ability to think positively about the care given to loved one and relationship with loved one at end of life.* |
| 1. Ability to talk about the deceased person without being overwhelmed |

| **Quality of life and general wellbeing** |
| --- |
| 1. Physical health and wellbeing |
| 1. Physical functioning i.e. being able to perform daily tasks |
| 1. View of one’s self |
| 1. Emotional wellbeing and meaning in life |
| 1. Mental health and wellbeing |
| 1. Financial security and material wellbeing /ability to address finances/ Ability to deal with social and financial insecurities and circumstantial factors |
| 1. Participation in work or recreational activities |
| 1. Learning, creativity and vitality |
| 1. Relationships with family and friends |
| 1. Relationship with health professional |
| 1. Satisfaction with home, neighbourhood and community environment |

| **Locus of Control** |
| --- |
| 1. Extent to which one perceives events and outcomes in life as within their control |
| 1. Extent to which one wants to have control over specific events and outcomes in life |
| 1. Extent to which one expects to have control over specific events and outcomes in life |

| **Identity and Belief Systems** |
| --- |
| 1. Impact on/re-emergence of sense of self |
| 1. Identifying/validating the positive consequences of loss |
| 1. Making sense of loss |
| 1. Impact on cultural identity |
| 1. Impact on spiritual identity |

Support

| **Interpersonal and Social Support** |
| --- |
| 1. Access to physical or practical support (e.g. managing deceased’s possessions) |
| 1. Access to material support |
| 1. Access to emotional support (e.g. experiencing warmth, empathy etc.) |
| 1. Access to guidance and positive feedback |
| 1. Participation in social activities |
| 1. Able to express feelings openly and honestly |
| 1. Take comfort, hope and strength from relationships with others (friends, family, professionals)* |
| 1. Feel understood and connected with others in similar situations* (e.g. access to support from those with shared experiences)/ help with connectedness and feeling less isolated |
| 1. Developing empathy with and helping others going through similar experiences* |
| 1. Difficult relationships and interactions with friends and family members* (Difficult to manage expectations of friends and family) |
