# Supplementary material for: Coping and wellbeing in bereavement: two core outcomes for evaluating bereavement support in palliative care
Source: BMC Palliat Care. 2020 Mar 12;19:29. doi: 10.1186/s12904-020-0532-4 (PMC7068975; doi:10.1186/s12904-020-0532-4)
Supplement: Supplementary file 3 — Additional file 3. Results of outcomes/dimensions mapping exercise. [file 12904_2020_532_MOESM3_ESM.docx]

**Additional File Three: Results of Outcomes/Dimensions Mapping Exercise**

|  | **Ability to Cope** | **Mental Health (and wellbeing)** | **Quality of Life** | **Social functioning and adjustment** | **Resilience** | **Grief Intensity** |
| --- | --- | --- | --- | --- | --- | --- |
| *Relationships with friends and family* | X | X | X | X | X | X |
| *Depression* |  | X | X |  |  | X |
| *Feelings of loneliness and emptiness* |  |  |  |  |  | X |
| *Ability to perform daily tasks* | X | X | X | X |  |  |
| *Anxiety* |  | X | X |  |  | X |
| *Ability to function as part of a family* |  |  | X | X |  |  |
| *Accessing emotional support if needed* | X |  |  |  |  |  |
| *Suicidal thoughts* |  | X |  |  |  | X |
| *Ability to take control/ look ahead and start to move forward with life* | X | X | X |  | X | X |
| *Ability to participate in social activities* | X | X | X | X |  |  |
| *Ability to express feelings openly and honestly* | X |  |  |  |  |  |
| *Sense of meaning and purpose* | X | X | X |  |  | X |
| *Feelings of blame, guilt, anger, bitterness, regret* |  | X |  |  |  | X |
|  | **Ability to Cope** | **Mental Health (and wellbeing)** | **Quality of Life** | **Social functioning and adjustment** | **Resilience** | **Grief Intensity** |
| *Understanding, acceptance, finding meaning in loss* | X |  |  |  |  | X |
| *Acceptance of grief experiences as normal* | X |  |  |  |  |  |
| *Accessing practical support if needed* | X |  |  |  |  |  |
| *Ability to find balance and channel grief* | X |  |  |  |  |  |
| *Optimism and hopefulness* | X | X | X |  | X | X |
| *Preoccupation with thoughts of the deceased* |  |  |  |  |  | X |
| *Ability to participate in work* | X |  | X | X |  |  |
| *Positive reminiscence and remembering of the deceased* | X |  |  |  |  | X |
| *Feeling understood by and connected with other bereaved people* | X |  |  |  |  |  |
| *Overwhelming thoughts and/or nightmares about loss* |  |  |  |  |  | X |
